# Experimental Zika Virus Inoculation in a New World Monkey Model Reproduces Key Features of the Human Infection

**DOI:** 10.1038/s41598-017-17067-w

**Published:** 2017-12-07

**Authors:** Charles Y. Chiu, Claudia Sánchez-San Martín, Jerome Bouquet, Tony Li, Shigeo Yagi, Manasi Tamhankar, Vida L. Hodara, Laura M. Parodi, Sneha Somasekar, Guixia Yu, Luis D. Giavedoni, Suzette Tardif, Jean Patterson

**Affiliations:** 1 Department of Laboratory Medicine, University of California, San Francisco, CA 94107 USA; 2 UCSF-Abbott Viral Diagnostics and Discovery Center, San Francisco, CA 91407 USA; 3 Department of Medicine, Division of Infectious Diseases, University of California, San Francisco, CA 94107 USA; 4 California Department of Public Health, Richmond, CA USA; 5 Texas Biomedical Research Institute, San Antonio, TX USA

## Abstract

A monkey model of Zika virus (ZIKV) infection is urgently needed to better understand transmission and pathogenesis, given its proven association with fetal brain defects in pregnant women and acute neurological illness. Here we experimentally infected 4 male marmosets with ZIKV (prototype 1947 African strain) and monitored them clinically with sampling of various body fluids and tissues for nearly 3 months. We show that the course of acute infection with ZIKV in these New World monkeys resembles the human illness in many respects, including (1) lack of apparent clinical symptoms in most cases, (2) persistence of the virus in body fluids such as semen and saliva for longer periods of time than in serum, and (3) generation of neutralizing antibodies as well as an antiviral immunological host response. Importantly, ZIKV-infected saliva samples (in addition to serum) were found to be infectious, suggesting potential capacity for viral transmission by the oral route. Re-challenge of a previously infected marmoset with a contemporary outbreak strain SPH2015 from Brazil resulted in continued protection against infection, no viral shedding, and boosting of the immune response. Given the key similarities to human infection, a marmoset model of ZIKV infection may be useful for testing of new drugs and vaccines.

## Introduction

Zika virus (ZIKV) is an infectious RNA flavivirus primarily transmitted to humans by the bites of *Aedes* spp. mosquitoes^1,2^. An outbreak of ZIKV began in Brazil in early 2015 and has since spread throughout South America, Central America, and the Caribbean, with autochthonous cases now being reported in the United States (Miami, Florida, and Texas). The rapid emergence of ZIKV in the Western Hemisphere is of particular concern given the proven association of viral infection with devastating fetal outcomes in pregnant women, including miscarriage and microcephaly^3^. Although the majority of ZIKV-infected individuals (~80%) are asymptomatic^4^, patients can present with a self-resolving acute illness consisting of fever, conjunctivitis, rash, and joint pain. Rarely, ZIKV has also been associated with neurological complications such as meningoencephalitis^5^ and Guillain-Barré syndrome^6^.

Although the primary mode of ZIKV transmission is via mosquito bite, it has also been shown that the virus has the capacity for sexual transmission^7^. Following an acute infectious episode, the virus can reside in semen for at least 3 months^8^. The virus has also been detected for at least 2 weeks after symptom onset in saliva and urine samples from acutely infected individuals^9^, although it is unknown whether the sampled body fluids were infectious. ZIKV transmission by blood transfusion from an infected donor has also been reported^10^.

To date, there have been several published mouse models of ZIKV infection; however, these have focused on studying ZIKV-associated complications in pregnant females such as fetal microcephaly^11–14^, and have required the use of immunodeficient animals with defects in interferon-related signaling pathways, likely due to absence of STAT2 cytokine inhibition of ZIKV in mice^2^. A viable non-human primate (NHP) model may thus better reflect the biology and pathogenesis of ZIKV in acute human infections. Investigations with NHP can also enable serial sampling and analyses of body fluids (e.g. urine, saliva, feces, and semen) that are impractical with rodent models.

Rhesus and cynomolgus macaque models of ZIKV infection are currently in development^15–26^. However, there are compelling reasons to consider the common marmoset (*Callithrix jacchus* ***)***, a New World monkey, as a useful alternative candidate model for ZIKV investigation. Common marmosets are known to have a high susceptibility to infection by a variety of pathogenic outbreak agents^27^, including Ebola virus^28^, Lassa virus^29^, and titi monkey adenovirus − a virus found to be associated with cross-species transmission to both monkeys and humans^30^. Related flaviviruses to ZIKV, including dengue virus (DENV) and West Nile virus (WNV) are known to cause productive infections in marmosets^31,32^. Furthermore, the recent detection of ZIKV in serum or saliva from wild marmosets from Brazil (26.7%, 4 of 15 animals tested)^33^ suggests that marmosets are a potential reservoir for maintaining Zika virus in endemic countries.

Here we present a marmoset model of acute ZIKV infection generated by inoculating 4 animals with ZIKV, followed by clinical monitoring and serial sampling for nearly 3 months. We sought to evaluate ZIKV infectivity, pathogenesis, persistence in infected body fluids and potential transmission risk, and production of neutralizing antibodies. The host response to acute ZIKV infection was also investigated by lymphocyte phenotyping, cytokine analyses and global transcriptome profiling of blood from experimentally infected animals.

## Methods

### Animal Ethics Statement

All animal studies were conducted at the Southwest National Primate Research Center (SNPRC), Texas Biomedical Research Institute (TBRI); molecular, viral, and transcriptome analyses of marmoset body fluids and tissues were conducted at University of California, San Francisco (UCSF). TBRI is accredited by the Association for Assessment and Accreditation of Laboratory Animal Care (AAALAC) International and operates in accordance with the NIH and U.S. Department of Agriculture guidelines and the Animal Welfare Act. The Institutional Animal Care and Use Committee (IACUC) and the Institutional Biohazards Committee (IBC) of the TBRI approved all marmoset experiments related to this study. All experiments were performed in accordance with relevant guidelines and regulations.

Marmosets were kept healthy and well-nourished with strict feeding protocols and close monitoring of their health status prior to the start of the study and during the entire study period. One week before inoculation, animals were transferred to the biosafety level-2 facility at the SNPRC and housed individually in cages specifically developed for marmoset work. As they are social animals in the wild, all marmosets had auditory, visual, and olfactory access to each other throughout the study. Marmosets were sedated and humanely euthanized by administration of a sodium pentobarbital solution by a licensed veterinarian at the TBRI.

### ZIKV propagation in cell culture

Vero cells were inoculated with the 1947 Uganda strain of ZIKV (passaged 147X in mouse brain and 3X in Vero cells) in the African lineage, which has been maintained at the Viral and Rickettsial Disease Laboratory (VRDL) branch of the California Department of Public Health. Viral supernatants for cell culture passaging and the generation of infectious stocks were subjected to 3 freeze-thaw cycles and clarified by centrifugation for 10 min × 4000* g*. After cells achieved 80–90% confluency, cell culture media were changed to maintenance media with 2% FBS and were inoculated with 100 µL of passaged viral supernatant. Viral replication was monitored over 14 days by visual inspection under light microscopy for cytopathic effect (CPE).

### Experimental ZIKV infection of marmosets

Four healthy adult male marmosets, averaging 2.1 years of age (range: 2.0–2.3 years) and 391.7 g (range: 332–453 g), were inoculated intramuscularly with 0.1 mL of a 1 × 10^5^ pfu/mL culture of the 1947 Uganda African lineage of ZIKV. Samples from an additional 4 male marmosets were used as matched controls for performing comparative gene expression studies by transcriptome profiling. All study marmosets were pre-screened for ZIKV antibody by neutralization and were found to be negative.

**Figure 1:**
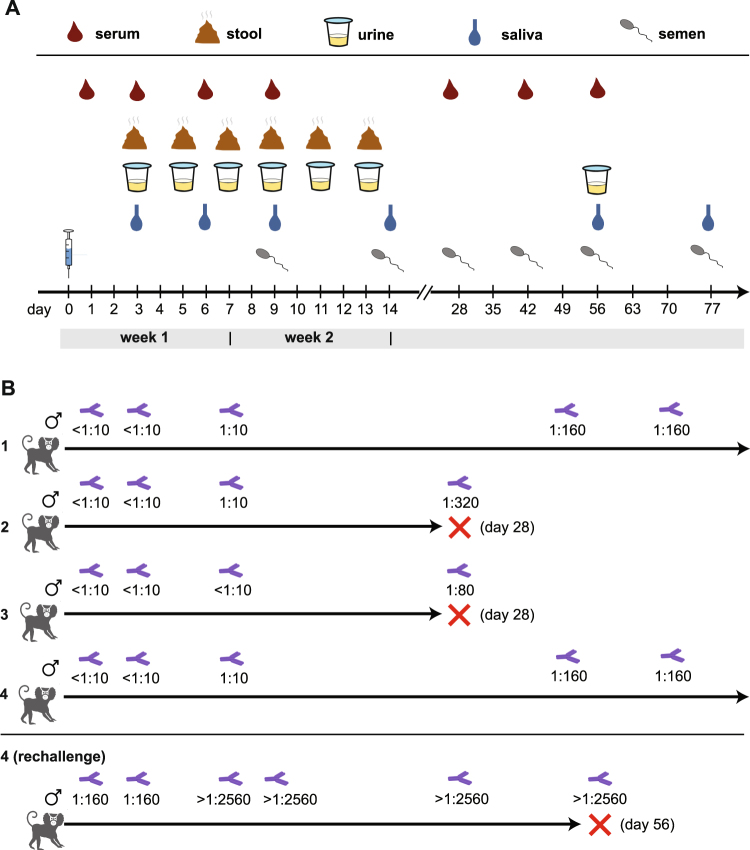
Study design and neutralizing antibody testing. (**A**) After intramuscular inoculation of ZIKV at day 0, samples (serum, stool, urine, saliva, and semen) are collected at predesignated time points. (**B**) Longitudinally collected serum samples from inoculated marmosets were tested at predesignated time points for ZIKV-specific neutralizing antibodies using a PRNT (plaque reduction neutralization test). The antibody titer as determined by PRNT at a given time point is shown below the icon. Three of the 5 marmosets were sacrificed after approximately 1 month (denoted by a red “X”) to assess viral persistence in tissues.

Animals were monitored daily for signs of clinical illness, with generalized sickness defined as a score of >4 (Supplementary Table 1). Specific monitoring was conducted for signs associated with ZIKV infection in humans, including rash, anorexia, conjunctivitis, diarrhea, malaise, and postural abnormalities associated with joint or head pain. Samples were collected from restrained, unsedated animals at predetermined time intervals. Animals were restrained for less than 10 minutes in a device specifically designed for short-term restraint of marmosets for sample collection purposes. For the 4 male marmosets, blood samples were collected via venipuncture on days 1, 3, 6, 9 and 28; voided urine and feces were collected on days 3, 5, 7, 9, 11, and 13; saliva was collected on days 3, 6, 9, and 14 by allowing the subjects to chew on a sterile cotton swab; semen samples were collected on days 9, 14, 28 by vibratory stimulation of the penis, using a modified FertiCare^TN^ medical vibrator unit (Multicept A/S, Denmark) (Fig. 1). Whole blood was collected in tubes containing RNA stabilization media (Biomatrica, Inc.) for transcriptome analysis. At day 28, 2 of the 4 male marmosets were randomly selected to be euthanized and their necropsy tissues examined for persistent ZIKV infection. The remaining two inoculated male marmosets were observed for an additional 7 weeks, with samples collected at weeks 7, 10 and/or 11 to evaluate long-term ZIKV persistence in body fluids. One of the remaining male marmosets was re-challenged with 0.25 ml of a 1 × 10^6^ pfu/mL culture of Brazilian ZIKV strain SPH2015^34^ 12 months after the first inoculation and followed clinically with serial sample collection until necropsy at day 56 (Supplementary Table 2).

### Measurement of infectious ZIKV titers by plaque assay

Plaque titration for quantification of infectious ZIKV was performed using Vero cells. 100 µL of serially dilutions of ZIKV from 10^−1^ to 10^−5^ was added to duplicate wells of 6-well plates containing a confluent monolayer of Vero cells, followed by incubation at 37 °C for 1 hr for adsorption. After adsorption, each well was overlaid with 2X Eagle’s Minimum Essential Medium in Earle’s balanced salt solution with 4% heat-inactivated FBS and 1.2% (w/v) Oxoid purified agar in water in a 1:1 ratio. The plates were maintained at 37 °C, 5% CO2 for 3 days, followed by 2^nd^ overlay with the same 2x medium with 0.08% neutral red and 2% Oxoid purified agar and in water in a 1:1 ratio. The 2^nd^ day after 2^nd^ overlay, plaques were counted and calculated in plaque forming units per mL (PFU/mL).

### Measurement of ZIKV RNA loads by quantitative RT-PCR

The course of infection in inoculated animals was monitored by determination of ZIKV RNA loads (expressed as RNA copies/mL) in serum, urine, saliva, stool, semen (collected in a conical tube at the time of ejaculation), and semen swabs (semen swabbed off of the penis and surrounding tissues immediately following ejaculation). Estimated ZIKV RNA loads were calculated by generation of a standard curve, followed by quantitative RT-PCR testing for 45 cycles using primers targeting the envelope gene (ZIKV-1086/ZIKV-1162)^35^. By standard curve analysis, the estimated limit of detection for the qRT-PCR assay is ~15 RNA copies/mL).

### ZIKV serological analysis by antibody neutralization

Plaque-reduction neutralization testing (PRNT) on longitudinally collected marmoset sera was performed by the California Department of Public Health. The protocol was similar to that used by the US CDC for confirmatory ZIKV testing in patients^36^. Briefly, 100 plaque forming units (PFU) of ZIKV (1947 Uganda strain or 2015 Brazilian SPH2015 strain, depending on the strain that was inoculated) were mixed with equal volumes of serial 2-fold dilutions of inactivated marmoset sera and incubated for 1 hr at 36 °C, followed by inoculation and adsorbing to a monolayer culture of Vero cells for 1 hr at 36 °C. After addition of 3 mL of 1% agar in Eagle’s Minimal Essential Medium (MEM), plates were placed in a 36 °C, 5% CO_2_ incubator ×3 days, followed by addition of 3 mL of 1% agar and 0.004% neutral red in Eagle’s MEM and another 1–2 days of incubation until plaques were formed. An 80% reduction of the number of plaques compared to positive control wells inoculated with virus-diluent mixtures was considered neutralization, with serum titers reported as the highest dilution exhibiting ≥80% reduction.

### Histology of ZIKV-infected marmosets

Samples of aseptically removed tissues were fixed in 10% neutral buffered formalin and embedded in paraffin for histology. Paraffin-embedded tissues were cut in 5 µm sections, de-paraffinized, and stained with hematoxylin and eosin (H&E) prior to visualization by light microscopy. Additional samples were freshly frozen in liquid nitrogen and kept stored in a −80 °C freezer until analyzed. Two board-certified veterinary pathologists independently evaluated the histologic sections.

### Lymphocyte phenotyping

Phenotypic characterization of marmoset peripheral blood mononuclear cells (PBMCs) was performed by multicolor flow cytometry using direct immunofluorescence. Aliquots of 100 μl of EDTA whole blood were directly incubated with antibodies for 20 minutes at room temperature; red blood cells were lysed with ammonium-chloride-potassium (ACK) buffer, and cells were then washed twice with phosphate-buffered saline (PBS) and fixed with 1.6% methanol-free formaldehyde before analysis in a CyAn ADP flow cytometer (Beckman-Coulter). The antibodies used for this analysis were conjugated to fluorescein isothiocyanate (FITC), Phycoerythrin (PE), Peridinin-chlorophyll-cyanin 5.5 (PerCP-Cy5.5), Phycoerythrin-cyanin 5.1 (PC5), Phycoerythrin-cyanin 7 (PC7), Pacific Blue, BD Horizon V500, Allophycocyanin (APC) or Alexa Fluor 700. Antibodies included in this study were: CD3 (clone SP34.2), CD4 (clone L200) and HLA-DR (clone G46.6/L243) from BD-Biosciences; CD14 (clone 322A-1 (My4), CD159a (NKG2A; clone Z199), CD20 (clone H299(B1)), CD335 (NKp46; clone BAB281) and CD337 (NKp30; clone Z25) from Beckman-Coulter; CD16 (clone 3G8), CD8 (clone HIT8a), CD86 (clone IT2.2) from Biolegend; and CD159c (NKG2C;clone 134522) from R&D Systems.

For analyses, lymphocytes were gated based on their characteristic forward and side scatter pattern, followed by T-cell selection using a second gate on the CD3-positive population. Thus, CD8 T cells were defined as CD8^+^/CD3^+^ and CD4 T cells as CD4^+^/CD3^+^. Natural Killer cells (NK) were defined as CD3^−^/CD20^−^/CD14^−^ and analyzed by the expression of NK cell markers CD16^+^, CD8, NKG2A, NKG2C, NKp30 and NKp46. B cells were defined as CD20^+^/CD3^−^/CD14^−^.

### Multiplex cytokine analysis of plasma

Plasma samples were analyzed for marmoset cytokines and chemokines on the Luminex 100 system (Luminex) using established protocols for New World primates^37^. The assay included evaluation of the following 21 analytes: GRO-α (CXCL1), interferon alpha (IFN-α), IFN-γ, interleukin-1 beta (IL-1β), IL-1 receptor antagonist (IL-1RA), IL-4, IL-8, IL-10, IL-12 p70, IL-15, IL-18, IL-22, monocyte chemoattractant protein 1 (MCP-1, CCL2), macrophage migration inhibitory factor (MIF), monokine induced by gamma interferon (MIG, CXCL9), macrophage inflammatory protein 1-alpha (MIP-1α, CCL3), MIP-1β (CCL4), regulated on activation, normal T cell expressed and secreted (RANTES, CCL5), tumor necrosis factor-alpha (TNF-α), soluble CD40 ligand (sCD40L), soluble intercellular adhesion molecule 1 (sICAM-1), and vascular endothelial growth factor A (VEGF-A).

### Transcriptome analysis

Four age-/sex-matched healthy marmosets were used as controls for the transcriptome analysis. All marmosets studied here were from a single colony, thus increasing genetic similarities and decreasing environmental bias. Technical bias in the whole transcriptome analysis was not observed by PCA (Supplementary Fig. 1).

Four hundred microliters of blood were drawn directly into RNAgard tubes (Biomatrica) for immediate RNA stabilization of intracellular RNA at collection. Total RNA was extracted using the Biomatrica Blood RNA Purification Kit (Biomatrica). The Ovation Human Blood RNA-Seq Kit (Nugen) was used to generate RNA-seq libraries from 100 ng of input per sample (as measured using the Invitrogen Qubit RNA HS Assay Kit) according to the manufacturer’s protocol. Libraries were sequenced as 100 base pair (bp) paired-end runs on a HiSeq 2500 instrument (Illumina).

Paired-end reads were mapped to the marmoset genome (*Callithrix jacchus* Ensembl version 3.2.1), using STAR 2.5^38^, and gene and transcript normalized counts were calculated by HTSeq version 0.6.0^39^. Differential expression of genes was calculated using linear modeling using the Bioconductor EdgeR software package version 3.12.2^40^ implemented in the R programming language. Genes were considered to be differentially expressed when their fold change was >±2, *p*-value <0.05, and adjusted *p*-value (or false discovery rate, FDR) < 0.1%. Pathway and network analyses of the transcriptome data were performed using Ingenuity Pathway Analysis (IPA) software (Qiagen).

### Data Availibility

Marmoset transcriptome data has been submitted to the public National Center for Biotechnology Information (NCBI) Gene Expression Omnibus (GEO) repository (accession number PRJNA315767).

## Results

### Experimental infection of marmosets with ZIKV

To investigate ZIKV infectivity in marmosets, and potential pathogenesis and persistence of virus in body fluids, including semen, we inoculated 4 healthy male marmosets intramuscularly with  0.25 mL of 10^6^ plaque-forming units (PFUs) of the 1947 Uganda prototype ZIKV strain MR766. The inoculation dose was chosen to be physiologic, comparable to the typical highest observed serum titers in patients with acute ZIKV infection^41^. Marmosets remained largely asymptomatic during the entire study period, with the exception of one male marmoset that exhibited drowsiness 2 days post-inoculation and had lost 7% of its body weight by day 5. However, this animal subsequently appeared alert and active and ate normally. No animal ever displayed a clinical score of > = 4, indicative of acute sickness, at any time during the study (Supplementary Table 1). Specifically, none of the other inoculated animals displayed anorexia, activity changes, or weight loss, and no subjects had fever, rash, conjunctivitis, diarrhea, or postural abnormalities suggestive of joint and/or muscle pain.

### ZIKV RNA in body fluids from experimentally infected marmosets

**Figure 2:**
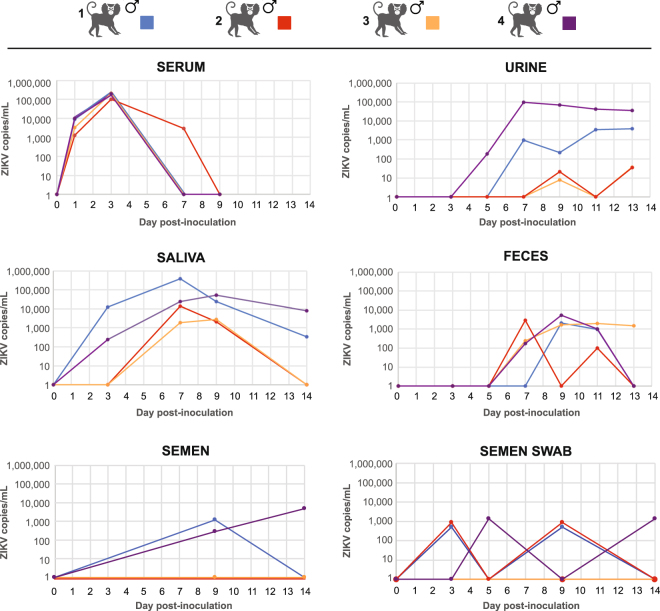
Viral loads in body fluids after acute ZIKV infection. The ZIKV load in copies per milliliter is plotted according to day post-inoculation. The line graph corresponding to each marmoset is displayed in a distinct color. Note that some lines are not visible in the serum graph due to similar viral load measurements that result in overlapping plots.

Serum, saliva, and urine samples were collected longitudinally at predesignated intervals (see Methods) for up to 14 days following inoculation. RNA loads of ZIKV (copies/mL) were estimated using quantitative ZIKV RT-PCR (Figs 1A, 2 Supplementary Table 2). A rapid rise and fall in ZIKV RNA, beginning at day 1 and returning to zero within 7–9 days, was observed in sera from all 4 inoculated male marmosets. Peak viremia was >10^5^ copies/ml at day 3 post-inoculation. In contrast to serum, ZIKV RNA loads in urine and saliva rose at later time points but persisted for longer periods of time, with peak viral production comparable to those observed in serum. Notably, at the end of the ~14 day collection period, 3 of 4 male marmosets (75%) and 2 of 4 (50%) were still shedding virus in the urine and saliva, respectively. Virus was also detected in the feces of inoculated animals beginning on day 5, albeit at much lower titers (10^2^–10^3^ copies/ml), and one animal (25%) continued to shed virus at day 13. ZIKV was also sporadically detected in semen and semen swabs in some, but not all, animals at a low level during the first 2 weeks following inoculation.

A ZIKV antibody neutralization assay by plaque reduction neutralization testing (PRNT), validated at the California Department of Public Health, was used to screen all experimentally infected marmosets for the development of neutralizing antibodies (Abs) to ZIKV (Fig. 1B). Importantly, all 4 male marmosets had negative pre-inoculation Ab titers of <1:10. Borderline ZIKV neutralizing antibody responses were detected in all 4 animals at a titer of 1:10 by day 6 post-inoculation, and positive titers ranging from 1:80–1:320 after week 4.

### Gross pathology and histology in ZIKV-infected marmosets

Two of the 4 male marmosets were euthanized at day 28 post-inoculation, respectively (Fig. 1B), for analysis of ZIKV pathology and persistence in tissues. No significant gross pathological lesions were observed in any of the post-mortem tissues. Salient histologic findings include mild-moderate nephropathy and vacuolization in hepatocytes associated with glycogen storage in the 2 euthanized animals. These histologic findings are common in healthy marmosets from this colony.

### ZIKV persistence in tissues and body fluids from experimentally infected marmosets

From the 2 male euthanized marmosets, nearly all of the necropsy tissues were negative for ZIKV by qRT-PCR, with the exception of detectable virus in lymph node tissue (3,680 copies/mg) from 1 male. To evaluate long-term persistence in body fluids, we also collected semen (6 and 10 weeks), semen swab (day 42), serum (6 and 10 weeks), urine (10 weeks), and saliva (11 weeks) from the 2 remaining marmosets. None of the samples collected after 6 weeks were positive for ZIKV by qRT-PCR.

### Viral infectivity from serum, saliva, and urine

Next, we sought to determine whether detected virus in body fluid compartments (e.g. serum, saliva, and urine) was infectious. We inoculated Vero cells with available ZIKV RT-PCR positive body fluids from infected male marmosets with RNA loads of 3.4 × 10^2^ to 1.9 × 10^5^ copies (Table 1). Viral cytopathic effect was observed after inoculation of 4 of 4 day 3 serum samples (each collected from an individual marmoset), and 4 of 4 day 7 saliva samples, but not from urine samples or day 14 semen samples.

**Table 1:** Cell culture of ZIKV from infected body fluids. Abbreviations: CPE, cytopathic effect; RT-PCR, reverse-transcriptase polymerase chain reaction; Ct, cycle threshold.

Primate ID	Collection day post-infection	Sample type	Volume of inoculum (μL)	Viral RNA copies of inoculum	Passage number	CPE present^*^	RT-PCR [Ct] of culture supernatant
1	3	saliva	100	1.24E + 03	0	++	+ [24.0]
1	3	serum	20	4.10E + 03	0	++	+ [18.3]
2	3	serum	20	2.08E + 03	0	++	+ [18.1]
3	3	serum	20	4.14E + 03	0	++	+ [19.4]
4	3	serum	20	4.00E + 03	0	++	+ [18.0]
1	7	saliva	100	3.88E + 04	0	++	+ [18.2]
2	7	saliva	100	1.41E + 03	0	++	+ [21.7]
3	7	saliva	100	1.92E + 02	0	++	+ [22.8]
4	7	saliva	100	2.40E + 03	0	++	+ [24.2]
4	7	urine	200	1.92E + 04	1^#^	—	—
1	13	urine	200	7.52E + 02	1^#^	—	—
4	13	urine	200	7.08E + 03	1^#^	—	—
1	14	saliva	200	6.86E + 01	0	—	—
4	14	saliva	200	1.57E + 03	0	—	—
4	14	semen	400	1.91E + 03	0	—	—
4	14	semen swab	400	5.60E + 02	0	—	—

* −, no CPE, +, mild-moderate CPE (<0–25%), ++, heavy CPE (>25%), reported from duplicate wells; cells were monitored daily for up to 14 days or until CPE was observed

#atypical cytotoxicity observed at passage 0, so cell culture supernatants passaged once in Vero cells.

### Cytokine and lymphocyte analyses

**Figure 3:**
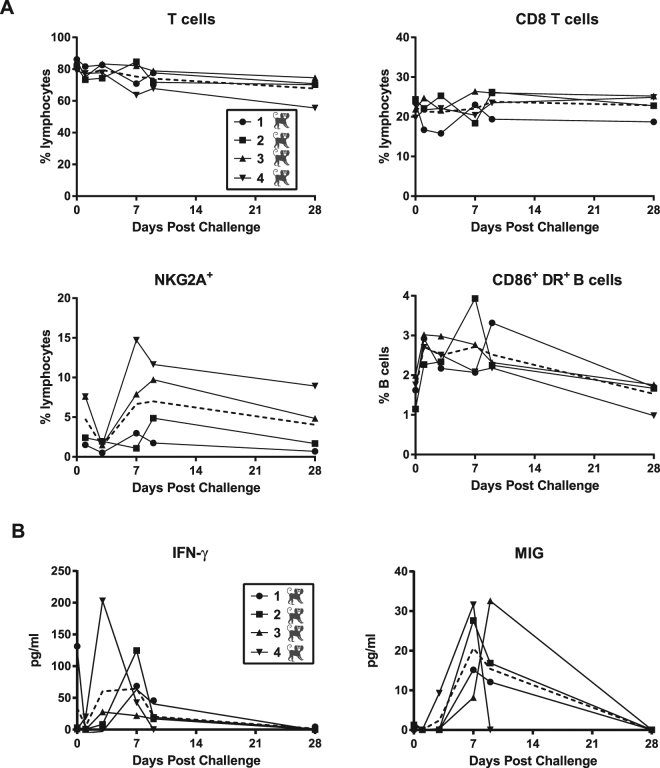
Changes in lymphocyte subsets and circulating cytokines after acute ZIKV infection. (**A**) Polychromatic flow cytometry was used to identify T cells (upper left), CD8 T cells (upper right), NKG2A + NK cells (lower left), and CD20 + B cells expressing activation markers CD86 and HLA-DR (lower right). (**B**) Increases in protein expression of interferon-gamma (IFN-γ) and monokine induced by IFN-γ (MIG) were detected using a Luminex assay.

Flow cytometry analysis of circulating lymphocytes in ZIKV-infected male marmosets showed no major changes for most of the lymphocyte subsets that were studied, including levels of T cells or CD8 T cells (Fig. 3A). However, we did observe an increase in the population of NKG2A + NK cells, which peaked by days 7–9 post-infection and returned to pre-infection levels by day 28 post-infection. There were also detectable increases in the levels of the NK activation markers NKp30 and NKp46 (data not shown). Interestingly, there was also a continuous up-regulation of the activation markers CD86 and HLA-DR on B cells during this acute period, returning to pre-infection levels by day 28 post-infection.

In parallel, we determined the plasma levels of 21 cytokines and chemokines with a validated Luminex assay^37^. The majority of these molecules were either below the limit of detection of the assay or did not change in a significant way after challenge with ZIKV (Supplementary Table 3). However, there was an increase over time in circulating IFN-γ and MIG (CXCL9, a monokine induced by IFN-γ), both members of the type II interferon signaling pathway, which peaked between days 3 and 9 post-infection, and returned to basal levels by day 28 post-infection (Fig. 3B). In contrast, circulating levels of IFN-α, representative of the antiviral type I interferon response, were always below the limit of detection (Supplementary Table 3).

### Whole transcriptome data analysis

The 4 asymptomatic ZIKV-infected male marmosets were sampled for whole blood transcriptome analysis at days 1, 3, 7, and 9 after post-infection, and were compared to 4 healthy uninfected male marmosets as controls. Two of the marmosets were followed up for 24 days post-infection and the remaining two marmosets were continually followed up for 42 and 64 days post-infection. The average sequencing depth was 25.0 M reads per sample (±13.9 M reads) (Supplementary Fig. 2). STAR/Cufflinks detected an average of 51.2% (±9.9%) of all Ensembl isoforms in each sample.

We examined whether there were any differentially expressed genes (DEGs) between the Zika-infected male marmosets at 1, 3, 7, 9, 42, and 64 days after post-infection and controls from their whole blood samples. For the first day post-infection (D1), 3 DEGs were found comparing Zika-infected against uninfected marmosets (Table 2). Three days post-infection (D3), 20 DEGs were found between the Zika-infected marmosets and controls, with 90% (n = 18) up-regulated and 10% down-regulated (n = 2). At seven days post-infection (D7), the difference in gene expression increased relative to the controls, with 43 DEGs found, 95% (n = 41) up-regulated and 5% (n = 2) down-regulated. By nine days post-infection (D9), 1,049 DEGs were found, with 67% (n = 706) up-regulated and 33% (n = 343) down-regulated. Two animals were sacrificed after day 9, and only 2 marmosets remained for follow up at day 42 and 64 post-infection, with 12 and 20 DEGs found respectively, but significance is uncertain given the low number of replicates.

**Table 2:** Number of differentially expressed genes (DEG) between ZIKV-infected and uninfected marmosets by day post-inoculation.

Comparison	Total DEGs	Up-regulated	Down-regulated
Day 1 versus uninfected	3	1	2
Day 3 versus uninfected	20	18	2
Day 7 versus uninfected	43	41	2
Day 9 versus uninfected	1049	706	343
Day 42 versus uninfected	12	5	7
Day 64 versus uninfected	20	9	11
Zika (all time points) versus uninfected	6	3	3

Gene ontology analysis revealed that 12 DEGs were shared between days 3, 7, and 9 in ZIKV-infected versus uninfected marmosets (Supplementary Table 4). All of the 12 DEGs were up-regulated, and there was an enrichment of terms related to the defense response to virus (GO:0051607), innate immune response (GO:0045087), and negative regulation of viral genome replication (GO:0045071). Notably, 6 of the 12 DEGs (MX1, MX2, ISG15, OAS2, OAS3, and GP2) were members of the type I interferon signaling pathway, whereas 1 DEG (GBP1) was a member of the type II interferon signaling pathway. Only one DEG, U3, a small nucleolar RNA, was shared among all sampled time points (1, 3, 7, 9, 42, and 64 days post-infection).

**Figure 4:**
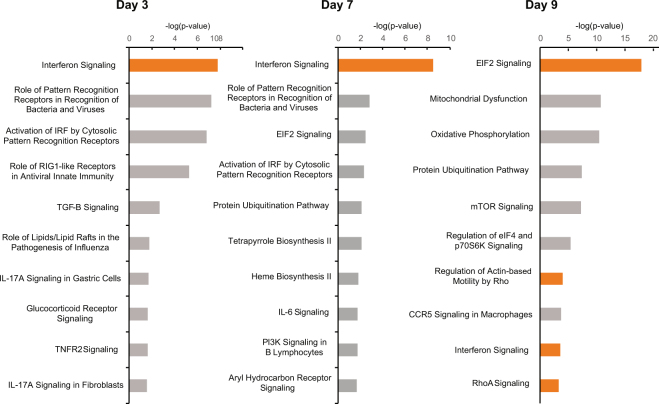
Top 10 canonical pathways associated with acute ZIKV infection by transcriptome profiling. Shown are the top 10 pathways at days 3, 7, and 9 post-inoculation, ranked by the negative log of the P-value of the enrichment score. The color scheme is based on Z-scores, with activation in orange and undetermined directionality in gray.

Canonical pathway analysis showed that the interferon signaling pathway, which regulates host resistance against viral infections, was the only pathway significantly up-regulated at all sampling time points: 3 (n = 20 pathways), 7 (n = 22 pathways), and 9 (n = 53 pathways) post-infection (Fig. 4). The type I interferon pathway was activated at days 3, 7 and 9, and type II interferon pathway was activated at day 9 (Supplementary Fig. 3). Pathways related to cell activation, including eIF2 signaling, actin-based motility by Rho family, and RhoA signaling pathways, were found to be significantly up-regulated only at D9. Pathway analysis at days 42 and 64 post-infection was attempted, but no pathway with significant up- or down-regulation could be predicted (data not shown).

### Rechallenge of a male marmoset with a contemporary Brazilian strain of ZIKV

To assess ZIKV infection after rechallenge with a different strain, we used the contemporary Brazilian SPH2015 strain to re-inoculate a male marmoset previously infected with the prototype 1947 Uganda strain. The marmoset remained asymptomatic, and all body fluid samples collected after infection, as well as post-necropsy tissues from organs, were negative for ZIKV by qRT-PCR (Supplementary Table 2). The baseline positive ZIKV Ab titer was 1:160 prior to and up to day 3 after rechallenge, but rose to >1:2560 by day 7.

## Discussion

In this study, we found that male marmosets inoculated with ZIKV did not develop signs of clinical illness, mimicking the approximately 80% of human infections that are asymptomatic^4^. Other features resembling human infections are (1) a brief period of viremia (<1 week), (2) persistent detectable ZIKV RNA in saliva and urine for at least 2 weeks following infection, and (3) sporadic detection in semen and stool. We also show that immunity elicited by the prototype 1947 Uganda strain (MR766) protects against subsequent infection with a contemporary outbreak strain (2015SPH). Taken together, these results indicate that the ZIKV marmoset model mimics important aspects of the human infection.

In ZIKV-infected marmosets, peak viral RNA loads in saliva and urine were comparable to those observed in serum, consistent with what has been previously documented in humans. Incorporation of these additional sample types is now part of many diagnostic and public health surveillance efforts. It is notable that ZIKV persistence in saliva and urine, unlike in blood, was not uniform, despite the fact that the marmosets were derived from a closed colony suggesting a population that was relatively homogeneous genetically. Thus, host and perhaps environmental factors likely play a role in determining the degree of ZIKV shedding in a particular individual, as has been shown for patients with acute ZIKV infection^42,43^. In 2 of the 3 marmosets that were euthanized after 1 month, we also found evidence of ZIKV persistence in lymph node tissue. Interestingly, acute ZIKV infection associated with lymphadenopathy has been described^44^, and viral persistence has been also shown in lymph nodes and cerebrospinal fluid samples from ZIKV-infected rhesus monkeys^17^.

Viruses isolated from serum and saliva samples from 4 of 4 ZIKV-infected male marmosets were capable of growth in cell culture, suggesting that these body fluids were potentially infectious. The possibility for ZIKV transmission through deep kissing has been raised in a recent case report describing sexual transmission of ZIKV^45^. However, although ZIKV has been detected in saliva^9,42^, no cases of human transmission through saliva have been documented to date. The male marmoset rechallenged with a contemporary strain of ZIKV did not show any evidence of active viral replication by qRT-PCR testing of serially collected body fluids and necropsy tissues. These results suggest that immunity elicited by the initial inoculation protected against subsequent infection; similar protection has been previously reported in a rhesus macaque model^18^. Notably, a marked increase in antibody titers was observed (from 1:160 to >1:2560) after rechallenge, suggesting non-sterilizing immunity and/or a secondary anamnestic response to ZIKV rechallenge.

We found by transcriptome profiling that ZIKV infection induces significant up-regulation of the type I interferon pathway at days 3, 7, and 9 post-infection (and the type II interferon pathway at day 9). However, parallel cytokine data show increases in protein expression of type II interferons (IFN-γ and MIG) and not type I at days 3, 7, and 9. ZIKV is known to inhibit the type I interferon pathway in human cells (but not mouse) by inducing STAT2 degradation by the proteasome^2^. Consistent with this report, we observed productive ZIKV infections of marmosets and up-regulation of both type I and II interferon signaling, but an increase in only type II interferon protein expression. It is possible that suppression of the type I interferon-related antiviral response in ZIKV infection in humans and non-human primates is rescued by type II interferon pathways.

Our study design used the prototype 1947 Uganda strain instead of the 2015 contemporary Brazilian strain. However, prior studies comparing the two have found that infection characteristics in rhesus macaques are similar^18,20^, and that the prototype 1947 Uganda strain is, similar to contemporary ZIKV strains, neurotropic and interferes with neurodevelopment^46^. Although less commonly used in biomedical research in the United States, the small size of marmosets does offer advantages in terms of easier housing and handling and decreased volume requirement for testing novel vaccines or therapeutics^28^. Surveillance data in wild marmosets also reveal that these monkeys may constitute a stable reservoir for the virus in the wild^33^, indicating that further investigations in this NHP model may have both clinical and public health relevance.

## Electronic supplementary material

Supplementary Information

Supplementary Table 2

Supplementary Table 3

Supplementary Table 4
