# Supplementary material for: Experimental Zika Virus Inoculation in a New World Monkey Model Reproduces Key Features of the Human Infection
Source: Sci Rep. 2017 Dec 7;7:17126. doi: 10.1038/s41598-017-17067-w (PMC5719425; doi:10.1038/s41598-017-17067-w)
Supplement: Supplementary file 1 — Supplementary Information [file 41598_2017_17067_MOESM1_ESM.pdf]

SUPPLEMENTARY INFORMATION

Experimental Zika Virus Inoculation in a New World Monkey Model Reproduces Key Features of the Human Infection

Charles Y. Chiu[123*], Claudia Sanchez San Martin[12], Jerome Bouquet[12], Tony Li[12], Shigeo Yagi[4], Manasi Tamhankar[5], Vida L. Hodara[5], Laura M. Parodi[5], Sneha Somasekar[12], Guixia Yu[12], Luis D. Giavedoni[5], Suzette Tardif[5], and Jean Patterson[5]

[1]Department of Laboratory Medicine, University of California, San Francisco, CA 94107, USA.

[2]UCSF-Abbott Viral Diagnostics and Discovery Center, San Francisco, CA 91407, USA.

[3]Department of Medicine, Division of Infectious Diseases, University of California, San Francisco, CA 94107

[4]California Department of Public Health, Richmond, CA

[5]Texas Biomedical Research Institute, San Antonio, TX

*Corresponding author: 185 Berry Street, Box #0134, UCSF China Basin, San Francisco, CA 94107, charles.chiu@ucsf.edu

Supplementary Table 1. Scoring system used for assessing clinical symptoms in marmosets experimentally infected with ZIKV.

Category	Scoring Criteria
Weight loss	0: no change from baseline 2: 5–10%↓ 3: 11–15%↓
Temperature / fever	0: no change from baseline 1: >2°F 2: >3°F 4: >5°F
Responsiveness	0: active 2: mild unresponsiveness, becomes active when approached 3: moderate unresponsiveness, lethargic (requires prodding), weakness 5: severe unresponsiveness, moribund
Recumbency	0: active 1: occasional prostration 2: persistent prostration but rises when approached 5: prostate
Activity:	0 = normal, active and alert 3 = abnormal, reduced activity
Dyspnea	0: normal breathing 3: labored 5: agonal
Petechiae or rash	0: none present 3: petechiae or rash present
Bleeding at venipuncture site	0: none 3: present
Bleeding other than venipuncture site	0: none 3: present
Nasal discharge	0: not present 3: visible discharge (copious)
Eyes	0: normal 1: discharge 3: partially closed 4: closed
Cubes eaten	0: ate 5-4 1: ate 4-3 2: ate 3-2 3: ate 2-1 4: ate 0-1 5: none
Stool	0: normal 2: diarrhea 4: reduced volume 5: no stool present
Fluid intake	0: drinking 2: reduced intake 3: not drinking
Dehydration (skin tent)*	0: test not done 1: 3 secs 3: 4 secs and up
TOTAL**	

* Skin tent test is performed during sedation time points.

** Total clinical score is determined by adding up all clinical scores by category. If total score is >20, animal is considered “terminally ill” and should be euthanized.

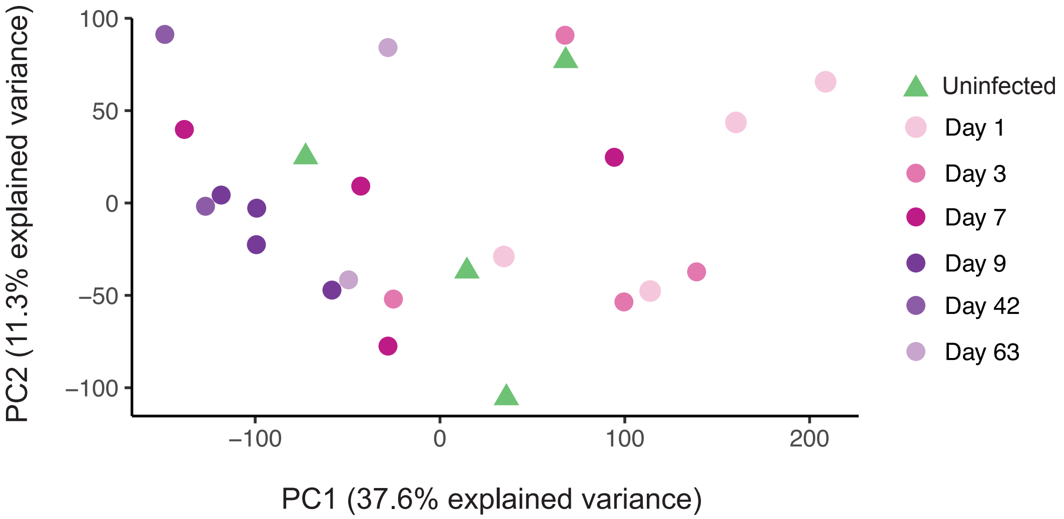

Supplementary Figure 1. Principal component analysis (PCA) of the gene expression profiles of ZIKV-infected marmosets and controls. A two-dimensional PCA plot comparing ZIKV-infected marmosets (circles, color-coded by day post-inoculation) and uninfected controls (green triangles) is shown. Gene expression profiles were obtained by whole blood transcriptome analysis. No apparent clustering suggestive of technical bias is observed.

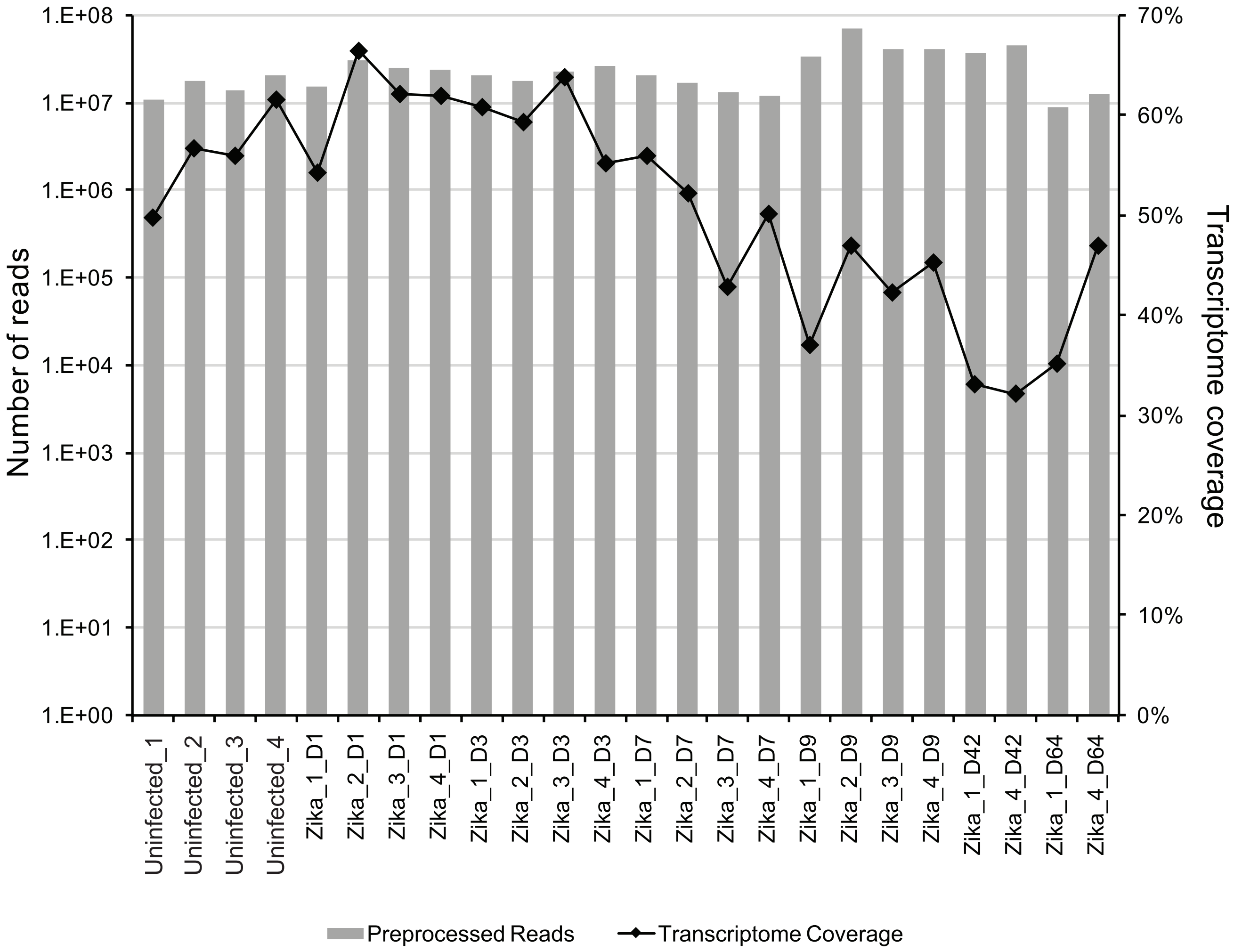

Supplementary Figure 2. Transcriptome coverage of whole blood samples from ZIKV-infected marmosets and controls. The bar graph shows the number of “preprocessed” reads, or reads remaining after removing low-quality (Phred score < 30) and short (length <100 bp) sequences.The line graph shows the transcriptome coverage as the percentage of gene isoforms with nonzero counts.

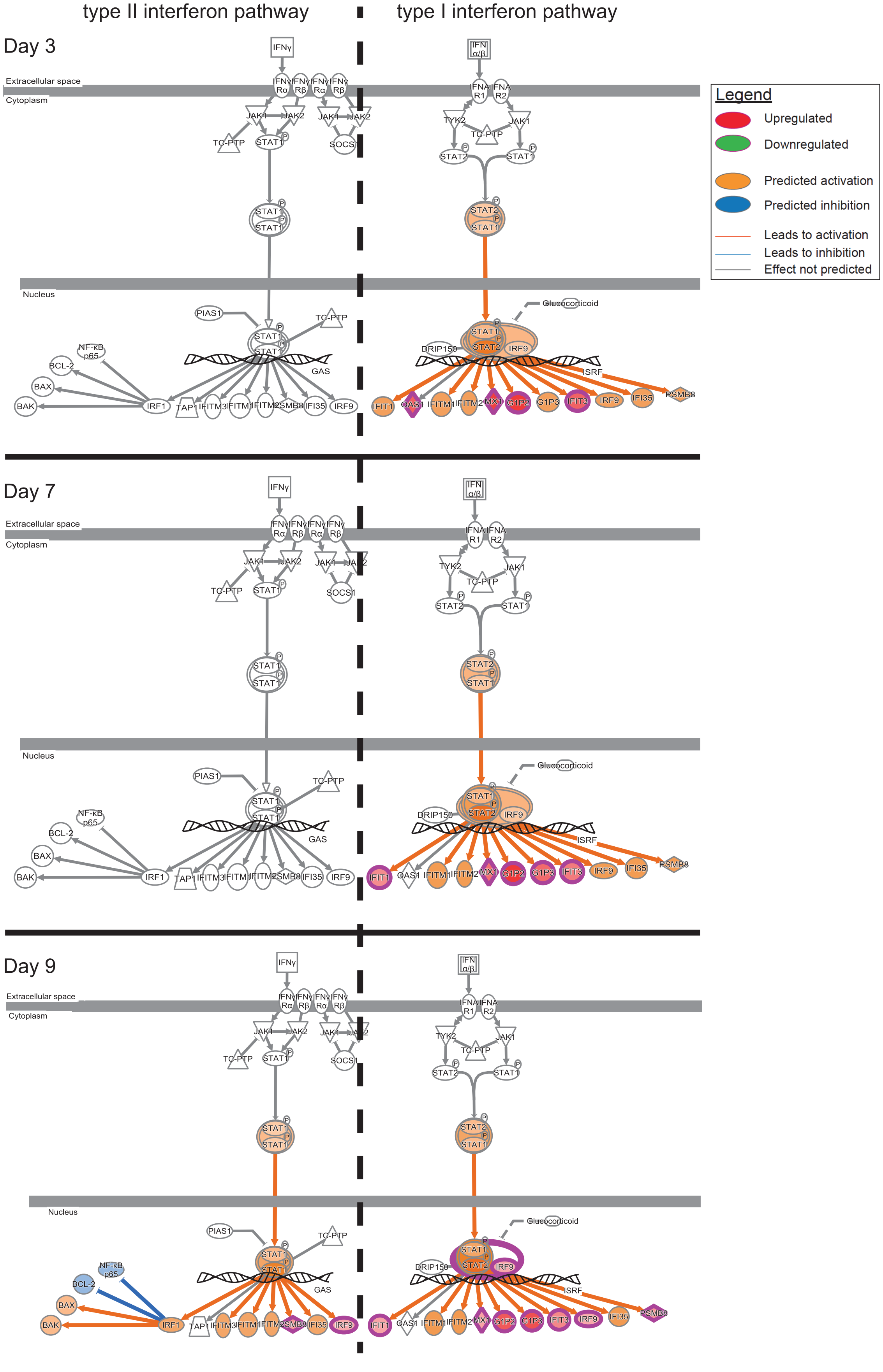

Supplementary Figure 3. Type I and II interferon pathways activated during acute ZIKV infection. Shown are transcripts associated with the type I and type II interferon pathways at days 3, 7, and 9 post-infection (red = transcript up-regulation; green = transcript down-regulation; orange = predicted activation, with darker shades of orange reflecting increased levels of activation; blue = predicted inhibition).
